# The Clinical and Public Health Challenges of Diabetes Prevention: A Search for Sustainable Solutions

**DOI:** 10.1371/journal.pmed.1002097

**Published:** 2016-07-26

**Authors:** Nicholas J. Wareham, William H. Herman

**Affiliations:** 1 MRC Epidemiology Unit, University of Cambridge, Cambridge, United Kingdom; 2 Departments of Internal Medicine and Epidemiology, University of Michigan, Ann Arbor, Michigan, United States of America

## Abstract

In an Editorial accompanying *PLOS Medicine*’s Special Issue on Diabetes Prevention, Guest Editors Nicholas Wareham and William Herman discuss some of the challenges for researchers and policy makers in developing effective and equitable solutions to the worldwide problem of type 2 diabetes.

The well-documented rise in the prevalence of type 2 diabetes (T2D) is a major global clinical and public health challenge, as described by Juliana Chan and Andrea Luk in their accompanying Perspective article [1]. Somewhat less attention has been paid to the potential for the rising global diabetes problem to widen health inequalities, and for the solutions that are put in place to tackle the problem to worsen or improve those inequalities. Inequity is at the heart of the diabetes problem, since 75% of cases occur in low- and middle-income countries [2]. The impact of diabetes on emerging countries will be particularly severe as the disease is chronic, expensive to treat, and tends to affect economically active people. The economic cost of dealing with the consequences of diabetes is not only a threat to health systems but is a far broader economic and social problem and thus a threat to future long-term sustainable development. Research aimed at diabetes prevention is therefore the focus of this special issue of *PLOS Medicine*.

The pattern of the incidence and prevalence of T2D between countries suggests that the disorder originates from a complex interaction between lifestyles, obesity, and innate susceptibility, driven by both genetic factors and developmental programming by maternal and postnatal nutrition. The major driving force behind the contemporary rise in prevalence is a rising incidence of disease driven by major secular changes in dietary and physical activity behavior. The T2D epidemic can be viewed at several different levels. At the individual level, the interplay between susceptibility factors and lifestyles is understood in broad terms, but our understanding at the molecular level is still limited. At the level of specific populations, more is understood about how changes in societal influences on diet and physical activity are driving the epidemic, but much of this understanding is restricted to developed countries. Finally, at the global level, there is a strong interconnection between the emergence of T2D and changes in the food supply and patterns of consumption, changes in transportation use, and related issues such as air pollution and climate change [3]. At this level, T2D can be seen as a clinical manifestation of a wider societal problem created by the undesirable consequences of rapid economic development.

Randomized controlled trials in people at high risk of diabetes clearly demonstrate that lifestyle interventions can result in an approximate halving of the risk of developing the disease, exceeding the apparent benefit of oral diabetes drugs [4]; this impact of behavior-change programs can be sustained over the long term [5]. These studies prove that T2D is preventable but do not directly provide information about how it should be prevented in real-world settings. Even in developed countries, efforts to translate such research interventions into practical programs have met challenges, with a large gulf persisting between efficacy and effectiveness, the gap between the health benefits that are achievable in a clinical trial as opposed to those that are realized in the real world [6]. As an illustration, in a research article in this issue, Laura Gray and colleagues report on a diabetes prevention trial carried out in the United Kingdom, and their findings indicate the importance of engaging and retaining people in such prevention programs [7].

Diabetes prevention programs that target high-risk individuals require an integration of efforts to test for prevalent undiagnosed diabetes, as well as so-called prediabetes, and are in essence large scale screening programs for hyperglycemia and diabetes. In the short term, integrated screening programs will increase costs as patients with newly diagnosed diabetes are identified alongside people with prediabetes who require monitoring and follow-up. In the longer term, this type of integrated program may be cost-effective, but this still remains to be demonstrated. Such approaches to prevention need to be focused on a relatively small group of people at high absolute risk. Attempts to provide individual-level prevention interventions to large groups of people at moderately elevated risk are not likely to be cost-effective [8]. There is also a concern that inequalities in health could be widened by individual-level interventions because, in general, more affluent people tend to be more likely to accept invitations for screening and treatment [9].

There is no universal recommendation on whether a country should or should not adopt such a clinical approach to diabetes prevention, as investment would need to be considered in competition with other clinical priorities. It is difficult to see how under-resourced health care systems could switch clinical investment to individual-level prevention when they are failing to meet the demands to provide systematic care and treatment to people with diabetes or its complications. In such settings, calls to screen for hyperglycemia run the risk of swamping health care systems that are already struggling to provide care. In the face of ageing populations, health care systems globally will struggle to cope with treating the estimated 642 million people with diagnosed diabetes anticipated by 2040 [2]. To countenance trying to provide individualized preventive interventions to a further half a billion people who by then will have prediabetes would put those health systems under unbearable pressure and would be an unsustainable proposition.

Thus, as a complement to individualized approaches to prevention, there needs to be a considerable scaling up of research into the societal determinants of diabetes, and evaluation of solutions that tackle the root causes of the problem, which are fundamental shifts in population-level dietary and physical activity behavior. As discussed by Martin White in a Perspective in this issue of *PLOS Medicine* [10], the nature of the evidence base supporting these population-level solutions will be fundamentally different from that which underpins clinical interventions, because randomized controlled trials are unlikely to be undertaken and most studies will be quasiexperimental or observational. This evidence base is beginning to emerge—as an example, the research article from Lindsey Smith Taillie and colleagues in this special issue presents an analysis of the effects of a tax on nonessential energy-dense foods enacted in Mexico [11].

At present, the evidence base for public health approaches to diabetes prevention is dominated by research from developed countries. However, it is likely that population-based approaches will be even more important in relatively resource-poor countries because health care systems will not be able to afford prevention programs targeting high-risk individuals. Yet the evidence base for population-based approaches to diabetes prevention in developing countries is limited and needs to be generated specifically in those contexts, as solutions cannot be exported from developed countries. Given the economic circumstances of the countries in which the diabetes epidemic is most pressing, there needs to be much greater investment in development of the evidence base for sustainable solutions that narrow inequalities, are economically affordable, support rather than hinder economic development, and can bring about the long-term changes in public health outcomes that are so urgently required.

## Abbreviations

T2D: type 2 diabetes
